# Corporate social Responsibility's impact on passenger loyalty and satisfaction in the Chinese airport industry: The moderating role of green HRM

**DOI:** 10.1016/j.heliyon.2023.e23360

**Published:** 2023-12-08

**Authors:** Chenxing Wang, Taiming Zhang, Rongzhi Tian, Ruogang Wang, Fahad Alam, Md Billal Hossain, Csaba Bálint Illés

**Affiliations:** a Changchun Tongtai Corporation Management Service Co., LTD, China; b Business School, The University of Edinburgh, China; c Institute for International Strategic Studies, Party School of the Central Committee of C.P.C, Beijing, China; d Southwest Forestry University, China; e School of Economics and Management, University of Science and Technology Beijing, Beijing, China; f Business Management and Marketing Department, School of Business and Economics, Westminster International University in Tashkent (WIUT), Tashkent 100047, Uzbekistan; g John von Neumann University, Doctoral School of Management and Business Administration, 1117 Budapest, Hungary

**Keywords:** Corporate social responsibility, Loyalty, Satisfaction, Green HRM

## Abstract

Corporate social responsibility has been extensively discussed and linked to the firm performance by the researchers. However, a significant research gap remains unexplored and that is measuring the association between corporate social responsibility, passenger satisfaction, and loyalty in the context of two international airports in China. This research also measures the moderating impact of green human resources management on the relationship between CSR, passengers' satisfaction, and loyalty. Data from two international airports in China were collected through a questionnaire. A total of 269 questionnaires were used for statistical analysis using Smart PLS 3.3. The findings from the statistical analysis revealed that corporate social responsibility in the airport affected passenger satisfaction and loyalty positively and significantly. Moreover, green human resource management in an airport plays a moderating role between corporate social responsibility, passengers' satisfaction, and loyalty. Overall, the study's findings enrich the literature on CSR, customer satisfaction, and loyalty, portray GHRM's role in the airport setting, and suggest practical indications for services industries. Discussions, limitations, and future recommendations are also given.

## 1 Introduction

The growing demands and low-cost mobility are some aspects that have sparked strong competition in the airport industry by offering different prospects and facilities to passengers [1]. For instance, Travel and tourism in China contributed US $876 billion to the country's GDP in 2018 [2]. From 2018 to 2028, it is estimated to increase by up to more than 10.3 % [2]. This progress is expected to accelerate more due to the Sports events, tourism, and other easy traveling programs planned by the Chinese government. Accordingly, it can predict a massive hike in passengers and tourists to Beijing Capital International Airport and Guangzhou Baiyun International Airport in China. Due to rising demand, the airport industry accounts for numerous environmental pollutants, negatively affecting public well-being [3]. Given this situation, where international airports concentrate on establishing policies to guarantee a pleasant experience for travelers, their corporate social responsibility (CSR) is gradually receiving more consideration as a component of that goal [4].

Airport shopping and the physical environment play a significant role in entertaining international passengers and other visitors by offering vital services such as retailers, duty-free stores, restaurants, and hotels which are becoming critical for the airport industry due to their valuable source of revenue [5]. In this sense, the role of entertainment and the physical environment is significant in the airport [6], where travelers and customers directly impact financial success, satisfaction, and loyalty. Ultimately, the airport sector significantly contributes to China's economic growth [7]. Consequently, Prentice and Kadan [8] noted the customary function of airports as means of transportation extended into entertainment and satisfaction, such as shopping and customer quality services. In this way, core sustainability challenges in airport sectors are frequently experienced by passengers and visitors. With such rise and diversification in airport sectors, the administration focuses on creating a pleasant passenger experience during their stay. Islam et al. [9] suggest that customer perception of satisfaction and loyalty begins with quality services and a green environment. Therefore, human resource management focuses on creating and maintaining a positive perception among passengers by providing psychological comfort [10].

In the present era, the airport industry has been particularly concerned with implementing Corporate Social Responsibility (CSR) as a strategic approach to achieve corporate goals [11], while also decreasing the detrimental effects of their activities on well-being and social environments [8,11]. A CSR policy integrates a company's social and environmental responsibilities with its stakeholders, and it significantly enhances passengers' positive feelings and responses [12]; however, till our deep observation, The present research is conceptualized to cover this research gap by demonstrating how CSR factors impact passenger satisfaction and loyalty. Furthermore, the present research also boosts the existing satisfaction degree by integrating state-of-the-art amenities and quality services added to airports.

Moreover, this research also considered green human resource management (GHRM) as a relevant strategy that links with CSR and can display its sustainable role in the airport industry. As Amrutha and Geetha [13] suggested, GHRM is a psychological factor that creates an environmentally friendly image among individuals. Therefore, incorporating GHRM into service design in airport sectors can provide passengers with a distinguishing emotional experience [11]. Furthermore, as suggested by Farooq et al. [14], airports serve as entry points that convey the initial impression of a country. Inexperienced passengers arriving in a country for the first time often form their first impressions based on their experiences with airport staff services and the physical environment. Hwang and Lyu [15] highlighted that the key purpose of a foreign tourist is to explore a new culture, standard of living, and environmental aspects. Therefore, integrating a GHRM into CSR in airport sectors can enhance passenger satisfaction and loyalty and possess a sustainable environmental impact.

Although corporate social responsibility and green human resource management have been identified as important factors in improving customer satisfaction and loyalty, it has not yet been examined from the perspective of China's international air sectors. Only a few scholars have observed the impact of corporate social responsibility on passengers' pleasure and well-being [16,17]. However, the study gap exists: It is a critical dispute addressed in this research, and the primary objective of this study is to investigate.

o To study the impact of corporate social responsibility on passenger satisfaction and loyalty.
o To analyze the influence of CSR on Passenger satisfaction affected by (moderator) GHRM.
o To analyze the influence of CSR on Passenger loyalty affected by (moderator) GHRM.
• The study makes a significant contribution to the field of airport management and corporate social responsibility (CSR) by investigating the impact of CSR on passenger satisfaction and loyalty in China's international airport sector. It also explores the moderating role of green human resource management (GHRM) in this relationship. This research addresses a crucial gap in the literature, shedding light on how CSR initiatives and GHRM practices can enhance passenger experiences, foster loyalty, and contribute to sustainable environmental impacts in the dynamic and rapidly growing Chinese airport industry.

## 2 Literature review

### 2.1 Corporate social responsibility

The corporate social responsibility concept has been widely discussed in the past. Carroll [18] believed that identifying its precise origin is challenging. Bowen [19] defined corporate social responsibility as "the obligations of businessmen to follow those policies and strategies, to undertake those decisions, or to pursue those courses of action that are beneficial in terms of our society's goals and values". After a while, the CSR notion has transformed from a humanitarian approach toward a business strategy that aids corporates in attaining a successful competitive edge [20]. Consequently, CSR is now perceived as a strategic approach to accomplishing business goals [12], while reducing the negative effects of corporate acts on natural and social environments [21]. CSR assimilates corporations' societal and ecological obligations to their stakeholders [8,11], which can empower customer responses and shape positive perceptions. In this regard, Hristov et al. [22] found that CSR initiatives can bring a sustainable advantage that can increase positive customer perception and psychological behavior such as satisfaction, positive mindset, and loyalty.

CSR literature is classified by the stakeholder theory [20]. A stakeholder is a person, group, or entity a business engages with to execute its objectives [9]. Ji, Tao, and Rim [23] proposed that business leaders respond to stakeholders' needs predominantly. Therefore, it is imperative to focus on their expectations and views as they can directly influence corporate performance. In this context, corporates need to link their CSR initiatives to the desires of their stakeholders and carry out CSR initiatives that are applicable to their business plan [24].

In this era, scholars have found that participating in CSR initiatives is linked to favorable outcomes. These embrace positive perceptions of corporate image and reputation [9, 25), strong affection [12], and higher satisfaction and loyalty among customers [25]. Additionally, some other favorable benefits, such as high sales, a green environment, a positive attitude, and psychological welfare, are reported by Raza et al. [24], specifically in the international airport industry, which is considered the busiest corporate sector. Previous studies have proposed several dimensions of CSR in different business sectors, revealing various influences on organizational and environmental perspectives [20,27]. Úbeda-García et al. [26] classified CSR into three major dimensions: (a) CSR society, (b) CSR customer, and (c) CSR employee. CSR society refers to the characteristics of a sustainable environment that can stimulate customer willingness (water waste and contamination, temperature, green environment, physical appearance). CSR customer refers to the emotional bond between the corporate and customers, which enhances customers' positive behaviors and attitudes, such as repeat purchase intentions, satisfaction, and loyalty. Lastly, a CSR employee indicates a commitment and deeds with a corporate that influence legal, ethical, and social obligations, increasing customer satisfaction [26]. For instance, Bhardwaj [27] researched environmental perspectives and suggested that CSR attributes enhanced stakeholders' and customers’ perceptions of psychological comfort and well-being. Similarly, Hayat et al. [28] shared some CSR successful performances in the organizational scenario, reflecting that CSR characteristics help to avoid unfavorable environmental activities, which boosts customer attraction. Moreover, previous scholars have found a significant relationship between different dimensions of CSR and its impact on different service sectors (see Table 1).

**Table 1:** Prior study on CSR dimensions.

Authors (Year)	CSR dimensions	Organization	Country	Findings
Choi and La [29],	ethical-legal component	Service sectors	USA	Findings indicate that CSR has significantly increased customer trust and loyalty.
Shin and Thai [30],	Environmental sustainability	Shipping Industry	South Korea	Empirical evidence supports that customers' perceptions of CSR significantly impact satisfaction and loyalty.
Irshad et al. [31]	social and environmental	Government and public financial sectors	Pakistan	Results found a positive impact of CSR on Customer Satisfaction and Loyalty.
Hossain et al. [32]	Medical facilities	health sectors	Bangladesh	A significant positive link between CSR and patient satisfaction and loyalty was found in healthcare sectors.
Latif et al. [20]	Values and Physical Environment	Hotel industry	Pakistan-China-Itlay	CSR can influence service quality, corporate image, and reputation, enhancing customer satisfaction.
Leclercq-Machado et al. [33]	Economical, Ethical, Legal	financial sector	Peru	The findings revealed that CSR enhances customer satisfaction and loyalty by mediating the impact of trust.

In some prior research, the role of CSR in different business sectors has been observed; however, the airport sectors still need to be incorporated into the perspective of China's international airports, particularly in considering multidimensional CSR attributes. Recently, in a study based on CSR linked with the Triple Bottom Line Theory, Shim et al. [34] proposed three dimensions for assessing CSR in airports; environmental, social, and economic. These dimensions incorporate some features of the airport's environment. However, Shim et al. [34] ignored some substantial aspects related to customer benefits, such as customers observing CSR gestures (green environment, electronic walkways, communications feasibilities, refreshments) to recognize latent desirable qualities (how compassionate a corporation is). They feel more connected to a corporation that practices CSR activities, which makes customers happy with their decision [20]. This study extends the existing scale proposed by Shim et al. [34], considering three major corporate social responsibility dimensions in the airport's physical environment: CSR society, CSR customer, and CSR employee.

CSR society is a strategy that encourages companies to behave in ways that benefit society and the environment rather than degrading it [35]. Niu et al. [36] observed that environmentally sensitive passengers seek sustainable and ecologically friendly goods and services. Accordingly, Ilkhanizadeh and Karatepe [4] highlighted a clean physical environment and clear routes are important for accessibility. In this regard, Prentice and Kadan [8] discovered that a sustainable layout was a significant factor in perceived quality services. Similar views were expressed by Okumus et al. [37], who claimed that passenger CSR perception is directly linked with a societal perspective; therefore, in the context of airports where thousands of passengers’ travel, an ecologically friendly layout is essential for their ultimate satisfaction and loyalty.

CSR customer refers to the strategy by which a corporation promotes a brand image and positive perception among customers for their own and society's well-being [38]. Scholars like Chang and Yeh [21] revealed that customer facilities could strengthen a corporation's reputation and add value to interior services linked with many favorable outcomes. Chang and Yeh [39] found that passengers' assessments of a facility significantly influence their attitude; consequently, better customer service generates a positive image. Prentice and Kadan [8] noted that in the airport scenario, customer CSR activities are a significant predictor which enhances passenger satisfaction and long-term attachment.

CSR employee refers to ultimate staff and their services to the customers, especially those frontline employees who are directly connected with customers [23]. Lee et al. [40] emphasized the importance of employee social responsibilities toward guests' satisfaction and loyalty in the hotel industry. Along this line, Park and Levy [41] point out that employee social responsibilities such as cleanliness and quality services can develop customers' positive perceptions regarding their ultimate care values. In the physical environment context, Loosemore and Lim [42] stated that employee cleanliness and environmentally friendly behavior could establish a significant brand image and positive feelings among customers. Prentice and Kadan [8] also found that the social responsibilities in the airport's physical environment (e.g., clean waiting rooms, refreshments, canteen shops, and other services) can positively influence customers' pleasure, satisfaction, and loyalty [8].

### 2.2 Customer satisfaction

Customer satisfaction (CS) is widely used in business industries. Prior scholars developed satisfaction metrics and examined their antecedents and implications [6, 45, 46); they believed long-term customer relationship is derived from their satisfaction. The concept and definition of customer satisfaction vary in the literature and corporate structure. However, all the conceptual frameworks concur that satisfaction implies the existence of an ultimate goal that every firm wants to achieve. CS is a customer's attitude, assessment, and reaction after a perceived experience of a firm product or service [43]. Sabir et al. [44] overviewed satisfaction in a cognitive context and suggested that it develops an emotional attachment to a company that leads toward ultimate loyalty. Another way to describe satisfaction is a person's assessment of a product or service based on their current emotive reactions [45,46]. More recently, Prentice and Kadan [8] observed that satisfaction is a positive emotion derived from assessing service quality with customer expectations. The previous literature also endorsed the influence of CSR on consumer attitude [9, 23, 46).

### 2.3 Customer loyalty

Customer loyalty (CL) often reflects consumer perceptions and assessments of a product or service. Singh and Sirdeshmukh [47] defined Customer loyalty as the behavioral intention of a person to maintain a steady and long-lasting connection with a firm or service provider. Scholars like Veloutsou [48] consider loyalty an emotional attachment that links high satisfaction and psychological comfort. CL has been identified as one of the significant goals of a corporation that ultimately enhances business outputs and develops a sustainable relationship with the customer [49]. Loyalty [50], slightly in a unique slant in the theory of expectancy-disconfirmation, proposes that individuals are attracted when they are amusingly pleased after confirming an experience. In the hotel industry, executives endeavor to attract customers by focusing on factors influencing customer decision-making. By so doing, airports need to be well-updated about the advanced trends in their industry to efficiently meet the needs and demands of progressively sophisticated travelers [51]. In the context of previous literature, passenger loyalty is a psychological reaction provoked by CSR activities in airport sectors [12]. Therefore, China airports were the first to recognize the significance of CSR and have become a flourishing research area in the airport industry.

## 3 Hypothesis development

The research demonstrates that consumers consider corporate social responsibility as an integral cue for assessing a corporate image [52]. Numerous studies have revealed that corporate social responsibility can induce customer psychological satisfaction and loyalty [23, 55). Latif et al. [20] conducted a study in the hospitality sector and found that social responsibilities develop positive feelings. Thus corporate social responsibility should be capable of eliciting customer satisfaction. A service setting's CSR comprises factors including cleanliness, feasible accessibility, standard and quality facilities, and proper functionality [53]; all of these factors connect to influence customer satisfaction and loyalty holistically [1,54]. Previous research shows that corporate social responsibility affects customers' sentiments and cognitions [20]. Given the importance of CSR in influencing passenger satisfaction and loyalty, we proposed that.

**Fig. 1:**
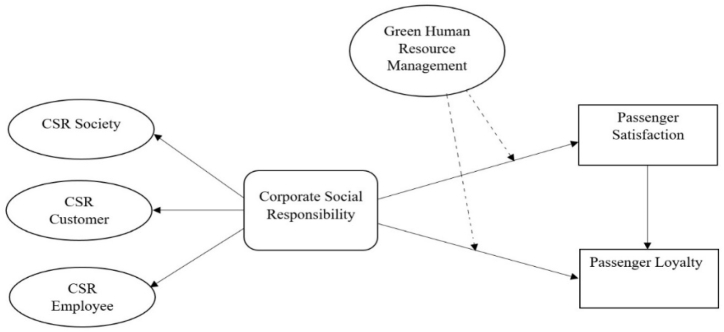
Researcher framework.

H1 Corporate social responsibility in the airport sector positively impacts passenger satisfaction (see Fig. 1).

H2 Corporate social responsibility in the airport sector positively impacts passenger loyalty (see Fig. 1).

Although scholars have found the importance of customer satisfaction in creating customer loyalty [55,56], however, empirical evidence revealed the impact of satisfaction on passenger loyalty is limited in the context of airports as compared to other sectors [18,46]. Ou and Verhoef [57] explored how feelings affect customer loyalty, clarifying that positive sentiments significantly influence customer loyalty. Additionally, Latif et al. [20] explored the influential role of consumer satisfaction on their loyalty in several aspects of the tourism industry. Still, no research has examined the impact of satisfaction in creating passenger loyalty in China airports; hence, this research formulates the following assumption.

H3 Passenger satisfaction significantly influences passenger loyalty (see Fig. 1).

### 3.1 Moderating role of green human resource management

Ahmad [58] defined green human resource management (GHRM) as “the use of human resource management policies, attitudes, and practices to promote sustainable use of organizational activities for the benefit of society, natural environment, and corporations. GHRM focuses on environmental-friendly HR practices that increase employee knowledge, awareness, and commitment to the concerns of sustainability [59]. Moreover, as Úbeda-García et al. [26] addressed, firms can use GHRM to cope with environmental issues and, consequently, develop a strong connection between stakeholders and customers regarding environmental concerns. Likewise, green human resource comprises unique traits of which the customers perceive a corporation's image as environment-friendly [58]. In recent times, air transportation has followed a sustainable approach as a corporate identity due to its implications on environmental protection [34]; consequently, it enhances passenger satisfaction and loyalty [60].

A prior study examined the significance of several components of GHRM in inspiring passengers to their specific destinations. For example, Ali et al. [45] discussed the Japanese visitors, what encouraged them to visit the United Kingdom, and what advantages they desired from their trip. They noticed that passengers are driven to seek fresh or unusual experiences in a different environment to explore a new destination. Likewise, Ali et al. [45] highlighted in their study that foreign visitors visited Thailand to spend a vacation and enjoy the host environment and environmental values. Most travel motivation studies have some aspects in common, such as a green environment, which significantly affects visitors’ feelings and behavior.

Understanding and identifying the distinctive qualities of a particular corporation is revealed by its sustainable environment. Despite this, many scholars have claimed GHRM is an influential factor for pre- and post-visitor behaviors [58]. However, a research gap still exists in examining the impact of green human resources on passenger satisfaction and loyalty in the airport service industry. The human resource department of a firm plays a critical role in creating a sustainable environment in international tourism. For instance, Farooq et al. [61] concluded that employee sustainable behavior and services are core elements that attract visitors and build a strong connection with the corporation, enhancing travelers' satisfaction. In a study on airport employee services, Lee et al. [62] confirmed the moderating role of GHRM aspects between passengers' perceptions of corporate image and their loyalty. If passengers experience green human resource services in the airport, they will display a more positive attitude toward their culture, which can increase satisfaction levels and loyalty. Consequently, passengers can perceive GHRM services as a sustainable strategy for society's benefit. Therefore, based on the above arguments, we proposed that.

H4 Green human resource management significantly influences the relationship between CSR and passenger satisfaction (see Fig. 1).

H5 Green human resource management significantly influences the relationship between CSR and passenger loyalty (see Fig. 1).

## 4 Research methodology

### 4.1 Research instrument

The item scale used to measure the dimension of corporate social responsibility (CSR society, CSR customer, CSR employee) was carried out by Ali et al. [45] study. The CSR was taken as a reflective construct. This approach is based on the existing research by Jeon et al. [63], who have taken the dimensions of CSR as reflective. Though these measurements were taken from various service industries (Hotels and organizations), the item scale they carried out ignored various important factors of the airport environment. Therefore, to establish a comprehensive items scale, the authors organized a group meeting after the literature review, including three (3) hospitality and tourism sector professionals and ten (10) frequent passengers. Consequently, fourteen (14) item scales connected to three dimensions of CSR of an airport were developed (See Appendix A): CSR society (4 items), CSR customer (5), and CSR employee (5 items). Each item was revised to reflect the study's context better. Moreover, the Kaiser-Meyer-Olkin and Bartlett's sphericity test were also assessed to measure the adequacy of factor analysis of these 14-item scales. The total measure of sample adequacy derived from the KMO test was 0.906 (>0.50), and 4441.197, df = 210, significant at p < .01, was the result of Bart's test to validate the suitability of the factor analysis. Items with a scale value less than 0.6 were neglected during the confirmatory factor analysis. All factor loadings of each item scale exceeded the standard criteria of 0.60 [64]. The three-factor solution (CSR society, CSR customer, CSR employee) accounted for 69.17 % of the variance. Another variable, Passenger satisfaction, was measured with Andaleeb and Conway's [65] six-item scale, which includes "I would be happy to consider this airport in the future". Passenger loyalty was operationalized by considering four items adapted from Chang and Yeh [39]. Example items include (This airport will be my first priority when I decide to travel). Finally, GHRM was measured using Renwick, Redman, and Maguire's [66] five-item scale. Such as, "The airport employees believed in environmental sustainability". All the items scale of these four variables was slightly rephrased to fit them according to the present study context; these items were rated on a 5-point Likert-type scale, ranging (from 1 = strongly disagree) to (5 = strongly agree). The questionnaire was written in Chinese (the National language) and English.

### 4.2 Data collection

The questionnaire was distributed among passengers through personal interactions at Beijing Capital International Airport and Guangzhou Baiyun International Airport, China from October 2022 to December 2022. At each airport, 150 questionnaires were distributed among international passengers using convenience sampling to obtain appropriate data samples in both airports. This method allowed us to include participants from diverse backgrounds, demographics, and travel experiences. This approach potentially represents meaningful insights from a wide range of individuals, enhancing the validity and credibility of our findings. We used this method because it is a popular and reliable research technique commonly used by scholars. The questionnaire survey method is cost-effective for contacting small and large groups. We can quickly collect data samples for statistical analysis [67]. This method is convenient as it involves selecting readily available individuals or elements for the sample. Those passengers who arrived and those who waited for their departure and expressed a willingness to participate in the research were targeted. To overcome language barriers and ensure that non-English-speaking respondents could provide accurate and meaningful answers, the scholars employed several measures during the data collection process. First, the scholar confirmed that the questionnaire instruments were written in both English and the native language commonly spoken by the participants in the selected region. Second, the questionnaire included clear instructions and, where necessary, provided accurate definitions and explanations of key terms to enhance respondent understanding. Third, a bilingual research assistant was involved in assisting participants and addressing any language-related concerns they faced during the survey. By implementing these measures, the scholar aimed to reduce language-related biases and ensure that respondents, regardless of their language proficiency, could provide accurate and meaningful responses. Out of 300 questionnaires, a total of 281 questionnaires were collected, of which only 269 were considered for further statistical analysis. The remaining incomplete questionnaire was neglected. By accounting for the 99 % confidence interval level, the 0.5 standard deviations, and 1 % margin of error, this data sample satisfies the recommended sample size of 205 respondents [45]. The absolute least sample size determined by Westland's [68] statistical software technique is 250 cases, which is the lower constraint for the sample size of our SEM model. The model is according to the latent variables along 29 items scales, with 0.80 statistical power and 0.5 significance. Hence, with 269 participants, our data sample meets the recommended minimum criteria for sampling adequacy [66]. The demographic characteristic of respondents is shown in Table 2.

**Table 2:** Participants’ profile.

Demographic characteristic		%
Gender	Male	61
	Female	39
Age	<24	19
	25–35	37
	36–45	23
	45>	21
Ethnicity	Asian/Middle Eastern	45
	European	22
	UK/American/Australian/African	19
	Others	14
Yearly travel frequency	1 - 2 times	47
	3 - 4 times	39
	More than 5 times	14
Traveling with	Friends/Colleagues	45
	Family members	17
	Alone	38

### 4.3 Statistical analysis

Common method variance should be tested when data are gathered by self-reported survey, specifically when the independent and dependent variables are gained from the same participant [69]. As a result, the authors adopted a few approaches to this problem, as given in the prior work. Initially, this study follows various extents for all measuring scales to gain active interest among participants. After that, experienced surveyors provided assurances of respondents' confidentiality and anonymity to encourage genuine responses. Finally, Harman's single-factor test was used to do a principle component factor analysis using all of the principal constructs. When a single factor appears from the factor analysis or one general factor explains most of the covariance between the measures, there is a common method bias [69]. First, we conducted a factor analysis in SPSS without rotation; the study produced a six-factor solution, which accounted for 68.78 % of the variation. The first factor explained only 31.53 % of the variance, showing that method bias was not a significant problem in this research. The inter-correlations (Table 4) indicated that all values are less than 0.9. Therefore, both tests suggest that there is no method bias in this study. The SPSS 28.0 software was run to assess the descriptive and reliability analysis of the gathered sample to evaluate the demographic characteristics and internal consistency. Then, using SmartPLS 3.3 software, we conducted a PLS analysis on the study model. We ran the measurement model in accordance with the suggested two-stage analytical techniques for SEM, and then we confirmed the structural equation model as suggested by Hair et al. [70]. PLS-SEM represents a well-substantiated method for estimating complex cause-effect-relationship models in management research [71]. To assess path coefficients and factor loadings, bootstrapping method with 5000 samples was run. The data normality was examined because structural equation modeling requires that the data not deviate from the assumption of normalcy. Kurtosis statistics ranged from −0.039 to 2.958, while skewness ranged was −1.937 to −0.402. Because some skewness values exceeded 2 while some kurtosis values exceeded 3, irreverent normality was considered in the data sample based on Kline's [72] standard criteria. Hence, partial least squares (PLS) were employed for statistical analysis.

**Table 3:** Validity and reliability.

Constructs Items		Loadings	AVE	CR
CSR society				
1	I believe the airport physical arrangements can help to minimize the negative influence on the natural environment.	0.917	0.678	0.886
2	The airport overall environment was clean and hygienic.	0.912		
3	The physical environment of airport can portray their country's aims toward a sustainable environment.	0.894		
4	The airport emphasizes the significance of social responsibilities toward society	0.902		
CSR customer				
	The airport you are now			
5	checks the standard quality of food/services provided to passengers	0.791		
6	respects passengers' rights beyond the legal requirements.	0.829		
7	Provide hygienic food and clean services.	0.904		
8	seems to be highly environmentally responsible.	0.789		
9	ensures that its services are equally accessible for overall passengers.	0.792		
CSR employee				
10	The airport employees display a positive attitude with professionalism and enthusiasm.	0.698		
11	The airport staff is well organized to offer standard service when required	0.787		
12	The airport employees were knowledgeable and friendly in helping passengers.	0.795		
13	I believe airport policies encourage workers to develop their skills.	0.784		
14	Overall, the airport employee display environmentally friendly behavior	0.811		
Satisfaction				
15	I am well-satisfied with my airport experience.	0.696	0.561	0.852
16	I would be happy to consider this airport in the future.	0.771		
17	I would gladly recommend this airport to others.	0.739		
18	Considering this airport, the quality of overall service was outstanding.	0.763		
19	I am really enjoying the overall environment of this airport	0.773		
20	I believe this airport is the most suitable place based on a sustainable environment.	0.752		
Customer loyalty				
21	I intend to choose this airport for traveling in the future.	0.901	0.635	0.869
22	This airport will be my first priority when I decide to travel.	0.883		
23	I will gladly recommend this airport if enquired by others.	0.895		
24	Overall, I believe that I am emotionally attached to the services of this airport.	0.886		
GHRM				
25	The airport employees believe in environmental sustainability.	0.681	0.564	0.862
26	I believe that employees' work behavior is favorable toward the airport's physical environment.	0.705		
27	The employees' environmental protection activities at this airport attract passengers.	0.774		
28	In this airport, Employees actively contribute to green environmental motivation efforts	0.785		
29	The airport implements green knowledge management which can enhance environmental protection.	0.803

**Table 4:** Discriminant validity.

Constructs	1	2	3	4	5	6
CSR society	0.811					
Passenger loyalty	0.685	0.797				
CSR employee	0.541	0.634	0.776			
CSR customer	0.458	0.597	0.564	0.801		
GHRM	0.402	0.527	0.611	0.538	0.751	
Passenger's Satisfaction	0.362	0.368	0.396	0.311	0.531	0.749

## 5 Results

### 5.1 Measurement model

Initially, we ran the measurement model to examine the convergent validity. Factor loadings, a CR (Composite Reliability), and AVE (Average Variance Extracted) were assessed. All the items' scales meet the recommended criteria of 0.6, as given in Table 3 [73]. The overall Alpha values and Composite reliability exceeded the recommended criteria of 0.700. The AVE values meet the minimum criteria of 0.5, which verifies convergent validity [71]. After that, discriminant validity was assessed. This refers to how much the measures do not reflect certain other variables. Table 4 demonstrates that each construct's square root of the AVE is greater than its corresponding correlation coefficient, indicating sufficient discriminant validity [71].

The Fornell and Larcker [74] criteria have recently come under criticism, with some claims that they cannot consistently identify the absence of discriminant validity in typical research scenarios. Henseler et al. [75] have proposed an alternate method the heterotrait-monotrait (HTMT) for evaluating discriminant validity. The HTMT value exceeded 0.85, suggesting that there is a discriminant validity issue [69]; however, Table 5 shows that all values are under 0.85, which satisfies the required criteria. Table 6 displays the weights of the 1st-order constructs on the chosen 2nd-order construct signifying that corporate social responsibility is a 2nd-order factor having three substantial 1st-order dimensions: CSR society, CSR customer, and CSR employee.

**Table 5:** Heterotrait-monotrait (HTMT).

Constructs	1	2	3	4	5	6
CSR society						
Passenger loyalty	0.792					
CSR employee	0.609	0.685				
CSR customer	0.436	0.442	0.662			
GHRM	0.631	0.501	0.481	0.693		
Passenger's Satisfaction	0.479	0.463	0.366	0.574	0.797

**Table 6:** Weights of 1st-order and 2nd second-order constructs.

2nd-order constructs	1st-order constructs	Weight	T-Value
CSR	Society	0.751	23.106**
	Customer	0.868	39.375**
	Employee	0.853	38.958**

### 5.2 Structural model

**Fig. 2:**
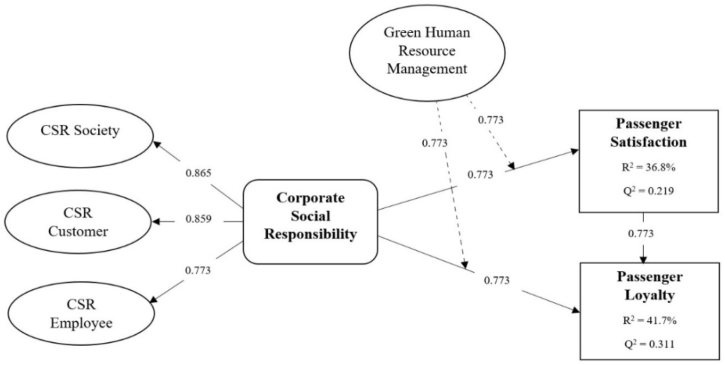
Structural model.

Hair et al. [70] recommended using a bootstrapping approach with a sample of 5000 to examine the R^2^ value, beta value, and corresponding t-values to evaluate the structural model. They also recommended that predictive relevance “Q^2^” and effect sizes “f^2^” should be reported along with the fundamental metrics. Initially, we observed the relationships between the targeted variables. CSR had a positive and significant impact on passenger satisfaction (β = 0.274; p < .01) and passenger loyalty (β = 0.219; p < .02). Additionally, satisfaction positively influences loyalty (β = 0.418; p < .02). Consequently, our study accepted hypotheses 1, 2, and 3 (See Table 7). In addition, CSR accounts for 36.8 % of the variance in satisfaction (R^2^ = 0.368), whereas CSR and passenger satisfaction demonstrate 41.7 % of the variance in loyalty (R^2^ = 0.417). The values of R^2^ (0.368 and 0.417) exceeded the recommended criteria of 0.26, as shown in Fig. 2, which specifies a substantial model [73].

**Table 7:** Structural analysis.

Hypotheses		Beta (β)	T Value	f^2^	Decision	p-value
H1	CSR > Passenger Satisfaction	0.274	4.372**	0.114	Accepted	0.00
H2	CSR > Passenger Loyalty	0.219	2.095**	0.103	Accepted	0.03
H3	Passenger Satisfaction > Passenger Loyalty	0.418	4.951**	0.125	Accepted	0.01
H4	CSR * GHRM > Passenger Satisfaction	0.519	6.518**	0.247	Accepted	0.00
H5	CSR * GHRM > Passenger Loyalty	0.386	5.651**	0.204	Accepted	0.00

(P < .05); (P < .01).

Moreover, we measured the effect size "f^2^". The p-value indicates the significance of the given relationships; however, it does not describe the effect size. Thus, understanding the facts and results would be an issue for readers. Therefore, it is important to report the substantive significance "f^2^″ as well as the statistical significance "p". Hair et al. [70] recommended variations in the R^2^ value must be examined.

This study considered Cohen's [76] criteria, which suggest 0.02, 0.15, and 0.35 are small, medium, and large effect sizes, respectively. All the relationships demonstrate medium effects, as given in Table 6. Moreover, Q^2^ demonstrates the effectiveness of data based on the model framework and the PLS constraints. A Q^2^ value larger than 0 indicates that predictive relevance exists in the model, while a Q^2^ value less than 0 indicates a lack of predictive relevance in the model. The endogenous variables satisfy the predictive relevance of Q^2^ as shown in Fig. 2.

### 5.3 Moderation analysis

This study considers GHRM as a moderating variable between CSR, passenger satisfaction, and passenger loyalty. By using the PLS product-indicator technique, moderating analysis was evaluated. To assess the moderating impact, corporate social responsibility and GHRM were multiplied to generate interaction constructs (CSR and GHRM) to predict satisfaction and loyalty among passengers. The calculated path coefficients of the moderating impact on passenger satisfaction (β = 0.519; p = .02) and passenger loyalty (β = 0.386; p = .02) were both significant, as shown in Table 7. This suggests that implementing the GHRM approach in airport sectors moderates the relationship between CSR activities in the airport, passenger satisfaction, and loyalty. Therefore, hypotheses 4 and 5 were confirmed and accepted.

## 6 Discussion

The study explored the importance of corporate social responsibility in improving passenger satisfaction and loyalty in airport sectors by testing the direct and moderating impact. Few studies have been conducted on the airport's physical setting. However, scholars have emphasized the necessity to look into developed countries' international airports and identify how to attract international visitors by providing quality services. Therefore, to the best of our knowledge, this study is the first attempt which examine the link between CSR in airports and passenger satisfaction and loyalty. Furthermore, this study examined the moderating role of GHRM in the relationship between CSR, passenger satisfaction, and loyalty. Five assumptions were investigated in light of the study's objectives and goals. This study accepted all the considered hypotheses. Our first two hypotheses were to check the impact of CSR on passenger satisfaction and passenger loyalty. The result reveals that CSR impact on passenger satisfaction and this result supports the social identity theory, suggesting that customers may identify with a firm that positively impacts society [77]. As a result, when passengers consider which airport to choose, they might pay close attention to CSR. Similarly, signaling theory supports our findings that CSR activities help convey a positive signal to the consumer mind and build a positive perception; consequently, it increases customer satisfaction levels and loyalty [20].

The third hypothesis of this study pertained to passenger satisfaction and passenger loyalty. The findings of this study closely align with previous research, providing evidence that higher passenger satisfaction leads to greater passenger loyalty [51]. The fourth and fifth hypotheses were related to how green human resource management moderates between CSR-PS and CSR-PL. The findings also demonstrate that GHRM positively moderates the relationship between CSR, satisfaction, and loyalty among passengers. The study verifies the positive impact of GHRM services on passenger perception. This study extends the GHRM literature by measuring the impact caused by green human services on environmental sustainability; In line with prior studies (28, 81), our results confirm that customers considered the implantation of the GHRM strategy as an environmentally friendly approach; accordingly, it increases the level of satisfaction and loyalty among the customers.

The major contribution that enriches the literature on hospitality management and environmental sustainability is to focus on the dimension of CSR role and its impact on passenger satisfaction and loyalty. According to study outcomes, this study endorses the theory of environmental psychology, which indicates the influential impact of the environment on cognitive states and individual behavior [78]. In the present study, corporate social responsibility activities in airports correspond to environmental aspects, passenger satisfaction represents emotions, and loyalty is a behavioral response. CSR's direct and moderating impact on passenger satisfaction and loyalty was empirically confirmed. Prior studies have explored the impact of airport background on passenger delight and happiness. Hence this existing study focused on the CSR activities of airports and their impact on passenger perception and behavior.

A few researchers have highlighted the role of CSR on customer satisfaction (20, 46, 83) and the significance of emotions on consumption attitude. However, determining the impact of CSR on passenger responses, specifically from the perspective of collective services, is still vital. Moreover, this study extends the research model by considering GHRM in airport sectors as a stimulating factor that may increase passenger satisfaction and loyalty. Font and McCabe [79] stated that sustainable services make the environment distinctive and noticeable, helping to portray corporate sectors as environmentally friendly. Scholars have discussed the idea of GHRM as a means to influence travelers' behavior. Till now, no studies have examined the impact of GHRM on passenger satisfaction and commitment in the airport sectors. The results provide empirical evidence for the moderating role of GHRM on the relationships between CSR, passenger satisfaction, and loyalty in the context of airport sectors.

Although there have been numerous studies on CSR in different sectors, including banking [80], restaurants [40], medical [81], and hotels [20], focus on airport CSR activities is rarely found. Therefore, this study followed the existing literature and added a newer items scale, resulting in a CSR scale with three dimensions and 14 items. Our results indicate that CSR is a 2nd order aspect along with three 1st order e.g. CSR society, CSR customer, and CSR employee, dimensions. Our study results revealed the complexity of CSR and its three significant factors, overall measured by visitors when they assess an airport's environment.

### 6.1 Managerial implications

Given the fierce competition in the airport industry, airports must build their distinct potential for attracting customers. However, according to Carroll's CSR dimensions, customer loyalty increases when they perceive the airport implementing desired CSR activities [53]. The results of this study should assist airport sectors in better understanding how air travelers view the corporate social responsibility initiative in the airport environment, which develops passenger loyalty by satisfying them. Previous studies have identified that customers may relate themselves to firms that influence environmental and social values [20,26,82,83]. Considering this argument in the airport industry, stakeholders should ensure overall emotional and psychological satisfaction among travelers.

When passengers experience positive feelings about CSR in airports, a positive image builds, which increases their psychological satisfaction. Consequently, stakeholders should focus on how CSR initiatives can generate favorable sentiments, extending passenger loyalty. Our findings support the implementation of the CSR approach raises revenues from airport sectors because satisfied passengers are more expected to choose the environmentally friendly physical environment. For example, Park et al. [84] surveyed behavioral intention among air passengers at three airports in South Korea, and findings show that social, economic, and environmental responsibilities and quality services significantly determine satisfaction levels. There was a unique link that maintained the loyalty intention of satisfied passengers. Hence, the literature reinforces the significance of customer choices of airports, and they may give paramount consideration to CSR.

Previous studies by Halpern and Mwesiumo [10] confirmed that CSR factors such as quality service, cleanness, facilities, and tangible and intangible green services significantly increase customer satisfaction and loyalty. Our study also aligned with the previous results: CSR dimensions are essential to an airport's performance and have a huge impact on passenger satisfaction and loyalty. Therefore, our study has practical implications. Particularly, CSR Societal, CSR customer, and CSR employee aspects should be continuously managed to enhance corporate success. Moreover, airports need to be well-equipped and astonishing, implementing environmentally friendly corporate activities that can generate positive emotions among passengers and visitors. In addition, higher positive emotions catch customer intentions (e.g., positive image and word-of-mouth, revisiting, repurchasing, high priority, and satisfaction) [20], which can lead to the success of an airport and raise its financial revenue.

Sustainability in the physical environment is vital for all international airports worldwide; incorporating GHRM in the airport service sectors can establish a unique and distinctive experience for the passengers. The findings suggest that GHRM is a significant factor in that uniqueness, which causes superior emotional engagement and satisfaction. Our results support prior research, which indicates that visitors' preference for a place in the global tourism and hospitality sector is based on various distinctive features. From the passengers' perception, CSR activities and a sustainable environment serve as the traveler's initial image for considering the arrival destination. Airports should therefore develop unique ways to offer services that can surprise and gratify travelers; hence, CSR activities and GHRM serve as a strategy that the air industry can implement to build a strong relationship with their passengers [85]. This approach will also help stakeholders better understand consumer behavior depending on how they view CSR initiatives to develop effective marketing strategies. The best model of this approach is carried out by Hamad International Airport, Qatar, the first-standard airport in the global rank based on passenger destinations in 2021. Hamad International Airport provides high-quality services along with a sustainable physical environment. The airport offers some standard services and retailers, along with a convenient passenger train that promptly transports passengers through different terminals [86]. The present study is the first to explore and validate the moderating impact of GHRM on passenger satisfaction and loyalty. Therefore, the outcomes reinforce that green human resources can be implemented as a marketing strategy to satisfy the existing passengers and enhance their loyalty to the specific airport.

### 6.2 Limitations and future directions

This study has a few limitations, which provide an opportunity for further consideration. First, corporate social responsibility is taken as a 2nd order construct with three dimensions (CSR society, CST customer, and CSR employee) for this study. This study considered a developed country China, to explore the influence of CSR on consumer-related outcomes in-depth; future research can consider other developing countries. Besides, Gestalt theory suggests that a person's attitude toward an environment depends upon social norms. Therefore, future research may extend this model with a more sustainable environmental approach in corporate sectors.

A noticeable limitation of this study was the small data which included 269 passengers, via used convenience sampling technique. In future studies, large data samples could be collected by applying different methodologies, which can provide more fascinating results. Furthermore, the authors used GHRM as a moderating variable in this study, and no mediating variable was used. Future studies can also determine other mediating and moderating variables in the relationship between CSR and customer response that may deliver interesting outcomes.

## 7 Conclusion

This paper investigates the impact of CSR on passenger satisfaction and passenger loyalty. The empirical results and findings suggest that there is a significant impact of CSR on passenger satisfaction and loyalty in the Chinese aviation industry. The result further shows that CSR is the prominent variable that is a vital prerequisite for passenger satisfaction. This paper also recommends various suggestions for airline managers regarding the problem-solving attitude in flight operations, keeping in mind that a satisfied traveler is a key factor in developing loyalty. Thus, airline personnel need to excel in order to make passengers satisfied. This is an important factor to survive in this competitive industry. Therefore, managers should devise strategies for passenger satisfaction. This study also highlights the role of green human resource management when integrated with CSR to satisfy passenger satisfaction and passenger loyalty.

The study findings offer valuable insights for future research. Firstly, a cross-cultural analysis could be conducted to explore the potential impact of CSR on passenger satisfaction and loyalty in different cultural contexts, providing a more comprehensive understanding. Second, as the global aviation industry continues to grapple with sustainability challenges and regulatory changes, future studies could investigate the impact of environmental policies and their alignment with airline CSR practices. Third, examining the role of CSR and its impact on passenger satisfaction and loyalty during crisis times, such as pandemics or natural disasters, would be pertinent, as these events can significantly affect the service industry. Additionally, it is essential to consider the evolving landscape of the airport industry, where factors such as technological advancements, changing consumer preferences, and global events (e.g., festivals, and international events) can have a substantial impact on passenger behavior. Future research should take into account these dynamic variables and assess how CSR strategies can adapt to address new challenges and opportunities. Addressing these research gaps can deepen our understanding of how CSR can enhance passenger satisfaction, foster loyalty, and maintain competitiveness in the airport and other service industry businesses.

## Data availability statement

Data will be made available on request.

## Funding

This research received no external funding.

## Ethics statement

This study was approved by the review and judgemental ethics of the research committee of the Hungarian University of Agriculture and Life Sciences and the approval number is 256,478/08.

## Institutional review board statement

Not applicable.

## Informed consent statement

Not applicable.

## CRediT authorship contribution statement

**Chenxing Wang:** Writing – original draft, Conceptualization. **Taiming Zhang:** Formal analysis, Data curation. **Rongzhi Tian:** Methodology, Investigation. **Ruogang Wang:** Software. **Fahad Alam:** Visualization, Validation. **Md Billal Hossain:** Writing – review & editing, Supervision, Methodology, Conceptualization, Funding acquisition, Validation. **Csaba Bálint Illés:** Writing – review & editing, Resources, Project administration, Validation, Funding acquisition, Data curation, Methodology.

## Declaration of competing interest

The authors declare that they have no known competing financial interests or personal relationships that could have appeared to influence the work reported in this paper.

## Figures and Tables

**Table:** 

Constructs	Items
CSR society	
1	I believe the airport physical arrangements can help to minimize the negative influence on the natural environment.
2	The airport overall environment was clean and hygienic.
3	The physical environment of airport can portray their country's aims toward a sustainable environment.
4	The airport emphasizes the significance of social responsibilities toward society
CSR customer	
	The airport you are now
5	checks the standard quality of food/services provided to passengers
6	respects passengers' rights beyond the legal requirements.
7	Provide hygienic food and clean services.
8	seems to be highly environmentally responsible.
9	ensures that its services are equally accessible for overall passengers.
CSR employee	
10	The airport employees display a positive attitude with professionalism and enthusiasm.
11	The airport staff is well organized to offer standard service when required
12	The airport employees were knowledgeable and friendly in helping passengers.
13	I believe airport policies encourage workers to develop their skills.
14	Overall, the airport employee display environmentally friendly behavior
Satisfaction	
15	I am well-satisfied with my airport experience.
16	I would be happy to consider this airport in the future.
17	I would gladly recommend this airport to others.
18	Considering this airport, the quality of overall service was outstanding.
19	I am really enjoying the overall environment of this airport
20	I believe this airport is the most suitable place based on a sustainable environment.
Customer loyalty	
21	I intend to choose this airport for traveling in the future.
22	This airport will be my first priority when I decide to travel.
23	I will gladly recommend this airport if enquired by others.
24	Overall, I believe that I am emotionally attached to the services of this airport.
GHRM	
25	The airport employees believe in environmental sustainability.
26	I believe that employees' work behavior is favorable toward the airport's physical environment.
27	The employees' environmental protection activities at this airport attract passengers.
28	In this airport, Employees actively contribute to green environmental motivation efforts
29	The airport implements green knowledge management which can enhance environmental protection.

## References

[1] Park S., Lee J.S., Nicolau J.L. (2020). Understanding the dynamics of the quality of airline service attributes: satisfiers and dissatisfiers. Tourism Manag..

[2] (2018). China inbound tourism development report. https://www.wta-web.org/wp-content/uploads/2022/03/China-Inbound-Tourism-Development-Report.pdf.

[3] Masiol M., Harrison R.M. (2014). Aircraft engine exhaust emissions and other airport-related contributions to ambient air pollution: a review. Atmos. Environ..

[4] Ilkhanizadeh S., Karatepe O.M. (2017). An examination of the consequences of corporate social responsibility in the airline industry: work engagement, career satisfaction, and voice behavior. J. Air Transport. Manag..

[5] Goyal R., Reiche C., Fernando C., Cohen A. (2021). Advanced air mobility: demand analysis and market potential of the airport shuttle and air taxi markets. Sustainability.

[6] Bezerra G.C., Gomes C.F. (2020). Antecedents and consequences of passenger satisfaction with the airport. J. Air Transport. Manag..

[7] Wu T.P., Wu H.C., Liu S.B., Hsueh S.J. (2018). The relationship between international tourism activities and economic growth: evidence from China's economy. Tourism Planning & Development.

[8] Prentice C., Kadan M. (2019). The role of airport service quality in airport and destination choice. J. Retailing Consum. Serv..

[9] Islam T., Islam R., Pitafi A.H., Xiaobei L., Rehmani M., Irfan M., Mubarak M.S. (2021). The impact of corporate social responsibility on customer loyalty: the mediating role of corporate reputation, customer satisfaction, and trust. Sustain. Prod. Consum..

[10] Halpern N., Mwesiumo D. (2021). Airport service quality and passenger satisfaction: the impact of service failure on the likelihood of promoting an airport online. Research in Transportation Business & Management.

[11] Han H., Lho L.H., Kim H.C. (2019). Airport green environment and its influence on visitors' psychological health and behaviors. Sustainability.

[12] Kim Y., Lee S.S., Roh T. (2020). Taking another look at airline CSR: how required CSR and desired CSR affect customer loyalty in the airline industry. Sustainability.

[13] Amrutha V.N., Geetha S.N. (2020). A systematic review on green human resource management: implications for social sustainability. J. Clean. Prod..

[14] Farooq M.S., Salam M., Fayolle A., Jaafar N., Ayupp K. (2018). Impact of service quality on customer satisfaction in Malaysia airlines: a PLS-SEM approach. J. Air Transport. Manag..

[15] Hwang J., Lyu S.O. (2020). Relationships among green image, consumer attitudes, desire, and customer citizenship behavior in the airline industry. International Journal of Sustainable Transportation.

[16] Chang Y.H., Yeh C.H. (2016). Managing corporate social responsibility strategies of airports: the case of Taiwan's Taoyuan International Airport Corporation. Transport. Res. Pol. Pract..

[17] Kim S., Hwang J. (2023). Airline CSR and quality attributes as driving forces of passengers' brand love: comparing full-service carriers with low-cost carriers. Sustainability.

[18] Carroll A.B. (1999). Corporate social responsibility: evolution of a definitional construct. Bus. Soc..

[19] Bowen H.R. (1953).

[20] Latif K.F., Pérez A., Sahibzada U.F. (2020). Corporate social responsibility (CSR) and customer loyalty in the hotel industry: a cross-country study. Int. J. Hospit. Manag..

[21] Chang Y.H., Yeh C.H. (2016). Managing corporate social responsibility strategies of airports: the case of Taiwan's Taoyuan International Airport Corporation. Transport. Res. Pol. Pract..

[22] Hristov I., Appolloni A., Cheng W., Huisingh D. (2022).

[23] Ji Y.G., Tao W., Rim H. (2022). Theoretical insights of CSR research in communication from 1980 to 2018: a bibliometric network analysis. J. Bus. Ethics.

[24] Kong Y., Antwi‐Adjei A., Bawuah J. (2020). A systematic review of the business case for corporate social responsibility and firm performance. Corp. Soc. Responsib. Environ. Manag..

[25] Le T.T. (2022). Corporate social responsibility and SMEs' performance: mediating role of corporate image, corporate reputation and customer loyalty. Int. J. Emerg. Mark..

[26] Úbeda-García M., Claver-Cortés E., Marco-Lajara B., Zaragoza-Sáez P. (2021). Corporate social responsibility and firm performance in the hotel industry. The mediating role of green human resource management and environmental outcomes. J. Bus. Res..

[27] Bhardwaj B.R. (2016). Role of green policy on sustainable supply chain management: a model for implementing corporate social responsibility (CSR). Benchmark Int. J..

[28] Hayat K., Jianjun Z., Ali S., Ageli M.M. (2022). Eco-advertising and ban-on-plastic: the influence of CSR green practices on green impulse behavior. Journal of the Knowledge Economy.

[29] Choi B., La S. (2013). The impact of corporate social responsibility (CSR) and customer trust on the restoration of loyalty after service failure and recovery. J. Serv. Market..

[30] Shin Y., Thai V.V. (2015). The impact of corporate social responsibility on customer satisfaction, relationship maintenance and loyalty in the shipping industry. Corp. Soc. Responsib. Environ. Manag..

[31] Irshad A., Rahim A., Khan M.F., Khan M.M. (2017). The impact of corporate social responsibility on customer satisfaction and customer loyalty, moderating effect of corporate image. City University Research Journal.

[32] Hossain M.S., Yahya S.B., Khan M.J. (2019). The effect of corporate social responsibility (CSR) health-care services on patients' satisfaction and loyalty–a case of Bangladesh. Soc. Responsib. J..

[33] Leclercq-Machado L., Alvarez-Risco A., Esquerre-Botton S., Almanza-Cruz C., de las Mercedes Anderson-Seminario M., Del-Aguila-Arcentales S., Yáñez J.A. (2022). Effect of Corporate social responsibility on consumer satisfaction and consumer loyalty of private banking companies in Peru. Sustainability.

[34] Shim J., Moon J., Lee W.S., Chung N. (2021). The impact of CSR on corporate value of restaurant businesses using triple bottom line theory. Sustainability.

[35] Tian Q., Robertson J.L. (2019). How and when does perceived CSR affect employees' engagement in voluntary pro-environmental behavior?. J. Bus. Ethics.

[36] Niu S.Y., Liu C.L., Chang C.C., Ye K.D. (2016). What are passenger perspectives regarding airlines' environmental protection? An empirical investigation in Taiwan. J. Air Transport. Manag..

[37] Okumus F., Sengur F.K., Koseoglu M.A., Sengur Y. (2020). What do companies report for their corporate social responsibility practices on their corporate websites? Evidence from a global airline company. Journal of Hospitality and Tourism Technology.

[38] Pérez A., Del Bosque I.R. (2015). Corporate social responsibility and customer loyalty: exploring the role of identification, satisfaction and type of company. J. Serv. Market..

[39] Chang Y.H., Yeh C.H. (2017). Corporate social responsibility and customer loyalty in intercity bus services. Transport Pol..

[40] Lee S., Han H., Radic A., Tariq B. (2020). Corporate social responsibility (CSR) as a customer satisfaction and retention strategy in the chain restaurant sector. J. Hospit. Tourism Manag..

[41] Park S.Y., Levy S.E. (2014). Corporate social responsibility: perspectives of hotel frontline employees. Int. J. Contemp. Hospit. Manag..

[42] Loosemore M., Lim B.T.H. (2017). Linking corporate social responsibility and organizational performance in the construction industry. Construct. Manag. Econ..

[43] Oliver Richard L. (1997).

[44] Sabir R.I., Ghafoor O., Hafeez I., Akhtar N., Rehman A.U. (2014). Factors affecting customers satisfaction in restaurants industry in Pakistan. Int. Rev. Manag. Bus. Res..

[45] Ali F., Kim W.G., Ryu K. (2016). The effect of physical environment on passenger delight and satisfaction: moderating effect of national identity. Tourism Manag..

[46] Pham T.S.H., Ahammad M.F. (2017). Antecedents and consequences of online customer satisfaction: a holistic process perspective. Technol. Forecast. Soc. Change.

[47] Singh J., Sirdeshmukh D. (2000). Agency and trust mechanisms in consumer satisfaction and loyalty judgments. J. Acad. Market. Sci..

[48] Veloutsou C. (2015). Brand evaluation, satisfaction and trust as predictors of brand loyalty: the mediator-moderator effect of brand relationships. J. Consum. Market..

[49] Rather R.A., Tehseen S., Parrey S.H. (2018). Promoting customer brand engagement and brand loyalty through customer brand identification and value congruity. Spanish Journal of Marketing-ESIC.

[50] Loyalty B. (2013). Study on the relationships among customer satisfaction, brand loyalty and repurchase intention. J. Theor. Appl. Inf. Technol..

[51] Akamavi R.K., Mohamed E., Pellmann K., Xu Y. (2015). Key determinants of passenger loyalty in the low-cost airline business. Tourism Manag..

[52] González-Rodríguez M.R., Martín-Samper R.C., Köseoglu M.A., Okumus F. (2019). Hotels' corporate social responsibility practices, organizational culture, firm reputation, and performance. J. Sustain. Tourism.

[53] Nikbin D., Hyun S.S., Iranmanesh M., Maghsoudi A., Jeong C. (2016). Airline travelers' causal attribution of service failure and its impact on trust and loyalty formation: the moderating role of corporate social responsibility. Asia Pac. J. Tourism Res..

[54] Barnes D.C., Krallman A. (2019). Customer delight: a review and agenda for research. J. Market. Theor. Pract..

[55] Bowen J.T., Chen S.L. (2001). The relationship between customer loyalty and customer satisfaction. Int. J. Contemp. Hospit. Manag..

[56] Kursunluoglu E. (2014). Shopping centre customer service: creating customer satisfaction and loyalty. Market. Intell. Plann..

[57] Ou Y.C., Verhoef P.C. (2017). The impact of positive and negative emotions on loyalty intentions and their interactions with customer equity drivers. J. Bus. Res..

[58] Ahmad S. (2015). Green human resource management: policies and practices. Cogent business & management.

[59] Mishra P. (2017). Green human resource management: a framework for sustainable organizational development in an emerging economy. Int. J. Organ. Anal..

[60] Türeli N.Ş., Durmaz V., Bahçecik Y.S., Akay S.S. (2019). An analysis of importance of innovatice behaviors of ground handling human resources in ensuring customer satisfaction. Procedia Comput. Sci..

[61] Farooq R., Zhang Z., Talwar S., Dhir A. (2022). Do green human resource management and self-efficacy facilitate green creativity? A study of luxury hotels and resorts. J. Sustain. Tourism.

[62] Lee S.S., Kim Y., Roh T. (2019). Modified pyramid of CSR for corporate image and customer loyalty: focusing on the moderating role of the CSR experience. Sustainability.

[63] Jeon M.M., Lee S., Jeong M. (2020). Perceived corporate social responsibility and customers' behaviors in the ridesharing service industry. Int. J. Hospit. Manag..

[64] Chin W.W. (1998). MIS Quarterly.

[65] Andaleeb S.S., Conway C. (2006). Customer satisfaction in the restaurant industry: an examination of the transaction‐specific model. J. Serv. Market..

[66] Renwick D.W., Redman T., Maguire S. (2013). Green human resource management: a review and research agenda. Int. J. Manag. Rev..

[67] Alam F., Yang Q., Rūtelionė A., Bhutto M.Y. (2023, May). Healthcare (Vol. 11, No. 11, P. 1537)..

[68] Westland J.C. (2010). Lower bounds on sample size in structural equation modeling. Electron. Commer. Res. Appl..

[69] Podsakoff P.M., MacKenzie S.B., Lee J.Y., Podsakoff N.P. (2003). Common method biases in behavioral research: a critical review of the literature and recommended remedies. J. Appl. Psychol..

[70] Hair J.F., Hult G.T.M., Ringle C., Sarstedt M. (2013).

[71] Gudergan S.P., Ringle C.M., Wende S., Will A. (2008). Confirmatory tetrad analysis in PLS path modeling. J. Bus. Res..

[72] Kline R.B. (2015).

[73] Chin W.W., Peterson R.A., Brown S.P. (2008). Structural equation modeling in marketing: some practical reminders. J. Market. Theor. Pract..

[74] Fornell C., Larcker D.F. (1981). Evaluating structural equation models with unobservable variables and measurement error. J. Market. Res..

[75] Henseler J., Ringle C.M., Sarstedt M. (2015). A new criterion for assessing discriminant validity in variance-based structural equation modeling. J. Acad. Market. Sci..

[76] Cohen J. (1988).

[77] Hu B., Liu J., Zhang X. (2020). The impact of employees' perceived CSR on customer orientation: an integrated perspective of generalized exchange and social identity theory. Int. J. Contemp. Hospit. Manag..

[78] Mehrabian A., Russell J.A. (1974).

[79] Font X., McCabe S. (2017). Sustainability and marketing in tourism: its contexts, paradoxes, approaches, challenges and potential. J. Sustain. Tourism.

[80] Platonova E., Asutay M., Dixon R., Mohammad S. (2018). The impact of corporate social responsibility disclosure on financial performance: evidence from the GCC Islamic banking sector. J. Bus. Ethics.

[81] Senay E., Landrigan P.J. (2018). Assessment of environmental sustainability and corporate social responsibility reporting by large health care organizations. JAMA Netw. Open.

[82] Du S., Bhattacharya C.B., Sen S. (2010). Maximizing business returns to corporate social responsibility (CSR): the role of CSR communication. Int. J. Manag. Rev..

[83] Rim H., Kim S. (2016). Dimensions of corporate social responsibility (CSR) skepticism and their impacts on public evaluations toward CSR. J. Publ. Relat. Res..

[84] Park E., Lee S., Kwon S.J., Del Pobil A.P. (2015). Determinants of behavioral intention to use South Korean airline services: effects of service quality and corporate social responsibility. Sustainability.

[85] Halpern N., Graham A. (2021).

[86] Rains T. https://www.businessinsider.com/best-airports-in-world-skytrax-rankings-doha-2022-6.

